# Lignin‐Derived Lightweight Carbon Aerogels for Tunable Epsilon‐Negative Response

**DOI:** 10.1002/advs.202401767

**Published:** 2024-05-07

**Authors:** Yunpeng Qu, Yunlei Zhou, Qiuyun Yang, Jun Cao, Yao Liu, Xiaosi Qi, Shan Jiang

**Affiliations:** ^1^ College of Physics Guizhou University Guiyang 550025 China; ^2^ Hangzhou Institute of Technology Xidian University Hangzhou 311200 China; ^3^ School of Geography, Geomatics and Planning Jiangsu Normal University Xuzhou 221116 China; ^4^ Key Laboratory for Liquid‐Solid Structural Evolution and Processing of Materials (Ministry of Education) Shandong University Jinan 250061 China; ^5^ School of Mechano‐Electronic Engineering Xidian University Xi'an 710071 China; ^6^ Jianghuai Advance Technology Center Hefei 230000 China; ^7^ State Key Laboratory of Intelligent Manufacturing Equipment and Technology Huazhong University of Science and Technology Wuhan 430074 China

**Keywords:** carbon aerogels, electromagnetic shielding, metamaterials, negative permittivity

## Abstract

Electromagnetic (EM) metamaterials have garnered considerable attention due to their capacity to achieve negative parameters, significantly influencing the integration of natural materials with artificially structural media. The emergence of carbon aerogels (CAs) offers an opportunity to create lightweight EM metamaterials, notable for their promising EM shielding or absorption effects. This paper introduces an efficient, low‐cost method for fabricating CAs without requiring stringent drying conditions. By finely tuning the ZnCl_2_/lignin ratio, the porosity is controlled in CAs. This control leads to an epsilon‐negative response in the radio‐frequency region, driven by the intrinsic plasmonic state of the 3D carbon network, as opposed to traditional periodic building blocks. This approach yields a tunable and weakly epsilon‐negative response, reaching an order of magnitude of −10^3^ under MHz frequencies. Equivalent circuit analysis highlights the inductive characteristics of CAs, correlating their significant dielectric loss at low frequencies. Additionally, EM simulations are performed to evaluate the distribution of the electric field vector in epsilon‐negative CAs, showcasing their potential for effective EM shielding. The lignin‐derived, lightweight CAs with their tunable epsilon‐negative response hold promise for pioneering new directions in EM metamaterials and broadening their application in diverse extreme conditions.

## Graphical abstract

A cost‐effective method for fabricating lightweight carbon aerogels (CAs) without requiring strict drying conditions is introduced. By tweaking the ratio of ZnCl_2_ to lignin, CAs’ porosity can be controlled, creating a customizable 3D carbon network with inherent plasmonic traits. This yields a weakly and tunable *ε′‐*negative response (*ε′* ≈ −10^3^), ideal for efficient electromagnetic shielding.

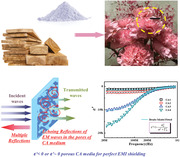

## 1 Introduction

Metamaterials have been rapidly evolving in recent years, revolutionizing various fields with their unique properties and functionalities.^[^ ^1^, ^2^, ^3^ ^]^ Intrinsic electromagnetic (EM) metamaterials are defined by the use of inherent material properties to create percolative composite structures.^[^ ^4^, ^5^, ^6^ ^]^ This approach enables the achievement of negative permittivity (*ε′* < 0), negative permeability (*µ′* < 0), or *ε′*‐near‐zero (ENZ) behaviors.^[^ ^7^, ^8^, ^9^ ^]^ These new types of EM media, characterized by extraordinary EM parameters, are poised to revolutionize material construction paradigms and transcend traditional EM response architectures.^[^ ^7^, ^8^, ^9^ ^]^ This is achieved through the meticulous design of microstructure units to tailor macroscopic physical field responses. Intrinsic EM metamaterials, a critical subset of metamaterials, exhibit remarkable EM response characteristics by engineering disordered structures at the sub‐wavelength physical scales.^[^ ^9^, ^10^, ^11^ ^]^ This design facilitates the regulation of interactions between radio frequency (RF) EM waves/fields and media, utilizing network‐structured composites.

Recent advancements in this field have been spearheaded by Fan et al., who pioneered the academic concept of using metal/ceramic percolative composite structures to attain negative parameters in the RF region.^[^ ^12^, ^13^, ^14^ ^]^ This method involves constructing a metal network within a porous ceramic matrix, effectively reducing the free electron concentration in metal‐based composites. Unlike the polarization response observed in traditional dielectric materials with positive permittivity, the epsilon‐negative response is a collective oscillation behavior of a large number of free charge carriers. This leads to a significant decrease in negative permittivity within the RF range, ≈4–6 orders of magnitude lower than that of bulk metals.^[^ ^12^, ^13^, ^14^ ^]^ Following this, Tsutaoka's team employed a similar technique, achieving negative parameters using both metal and ferrite for EM functions.^[^ ^15^ ^]^ The design of intrinsic EM metamaterials generally adheres to the academic principle of creating functional phase/matrix percolative composite structures, with a focus on elucidating percolation theory and exploring mechanisms behind *ε′*‐negative responses.^[^ ^16^, ^17^, ^18^ ^]^ Depending on the functional phases and matrix material categories, intrinsic EM metamaterials can be broadly classified into four groups: metal/ceramic, carbon nanomaterials/ceramics, carbon nanomaterials/polymers, and metal/polymers.^[^ ^19^ ^]^ Metals, known for their abundance of free electrons, high conductivity, intrinsic *ε′*‐negative response in optical frequencies, and sensitivity to EM wave/field variations, are commonly used in these metamaterials.^[^ ^20^ ^]^ Carbon materials, including graphene (GR), carbon nanotubes (CNT), carbon black (CB), and amorphous carbon, offer advantages such as moderate carrier concentration, high mobility, controllable geometric parameters, and chemical stability.^[^ ^21^ ^]^ These materials have increasingly been incorporated into metamaterial designs, as evidenced by CNT/polyvinylidene fluoride, amorphous carbon/Si_3_N_4_, and GR‐CB/CaCu_3_Ti_4_O_12_, all featuring adjustable negative parameters.^[^ ^22^, ^23^, ^24^ ^]^ However, the unstable interface matching between carbon materials and ceramic matrices leads to unsatisfactory frequency dispersion of the epsilon‐negative response.^[^ ^22^, ^23^, ^24^ ^]^ The approach of randomly constructing a 3D network with carbon functional phases limits the applicability of EM metamaterials under extreme working conditions.

In response to these limitations, researchers have recently introduced a new type of carbon‐based EM metamaterials: carbon aerogels (CAs).^[^ ^25^ ^]^ Unlike previous designs that relied on percolative structures composed of composite functional and insulating phases, CAs achieve negative permittivity through their intrinsic plasmonic state. They further regulate effective electron concentration via their porous structure, thereby enabling tunable epsilon‐negative responses. In contrast to metal‐based metacomposites, CAs not only exhibit excellent lightweight and chemical stability properties but also provide a novel method to modulate epsilon‐negative response in the RF region.^[^ ^26^ ^]^ Although CAs have demonstrated promising applications in areas such as supercapacitors, extreme heat insulation, and pollution treatment, the epsilon‐negative response mechanism of CAs remains underexplored since our team initially proposed them as structural units for EM metamaterials.^[^ ^25^, ^26^ ^]^ There is an urgent need for a universal design paradigm that focuses on the special structure and unique properties of CAs.

In this work, we introduce the first lignin‐derived CAs with a tunable epsilon‐negative response in the RF range. Lignin, a renewable and aromatic biopolymer, is an attractive alternative to fossil resources with toxicity and non‐degradability such as phenol‐formaldehyde resins.^[^ ^27^ ^]^ We present an efficient, low‐cost method to fabricate CAs without requiring rigorous drying conditions. This technique enables a tunable and weakly epsilon‐negative response through adjustable porosity of CAs. Additionally, we have conducted EM simulations to evaluate the theoretical EM shielding performance. This research opens up new avenues in the field of EM metamaterials and offers environmentally friendly, cost‐effective applications for CAs.

## 2 Results and Discussion

### 2.1 Composition and Microstructure of Porous Carbon Aerogels

**Figure 1:**
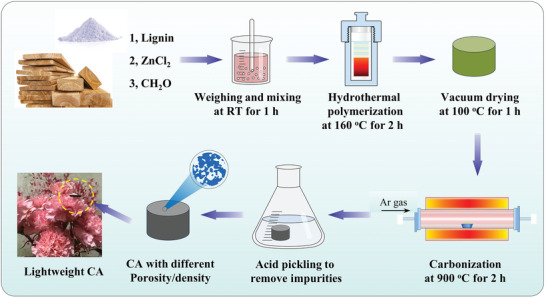
Stepwise preparation of lignin CAs.

Annually, the pulping process generates ≈50 million tons of technical lignin, a by‐product typically burned as a low‐value fuel, with only ≈2% utilized in specialty products.^[^ ^28^ ^]^ This underutilization is mainly attributed to lignin's low reactivity and its random, branched structure, making it challenging to polymerize into lignin formaldehyde resins without activation or the addition of phenols. Here, we brought up a simple and effective method to convert lignin into lightweight CAs with adjustable density and porosity, as illustrated in **Figure** **1**. The conductive carbon functional phase can provide good carrier transport characteristics, which is conducive to realizing the plasma oscillation effect, and then designing metacomposites with an epsilon‐negative response. Therefore, whether it is CAs or GR, CNTs, or other amorphous carbon, it can be a good candidate to build metamaterials. This method is also a universal and effective approach.

**Figure 2:**
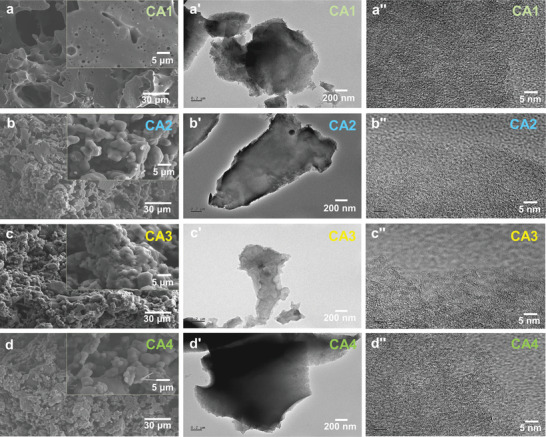
a–d) SEM images, a'–d') TEM, and a”–d”) HRTEM maps of CAs with varying densities.

Through hydrothermal polymerization and carbonization, we successfully produced lignin‐derived CAs. Precise control over the ZnCl_2_/lignin ratio allowed for the tuning of porosity, resulting in the formation of 3D carbon networks with adjustable effective electron concentration. The microstructures of the CAs are depicted in **Figure** **2** (more details can be found in Figure S1; Supporting Information), showing samples CA1 to CA4 all carbonized at 900 °C and exhibiting a similar degree of graphitization (Figure 2a″–d″). Since all CAs share a similar framework and porous structures, their dielectric responses are uniformly influenced by their interfaces and geometric factors, with porosity/density being the primary variable.

**Figure 3:**
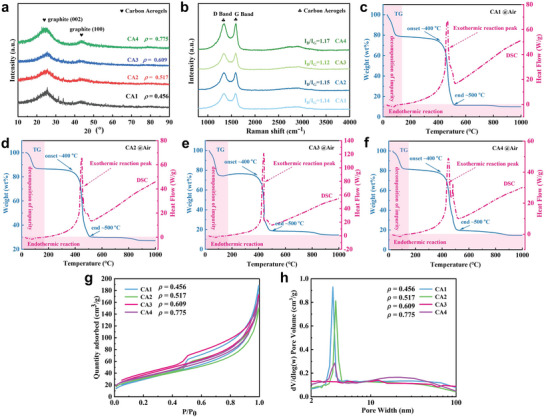
a) XRD patterns, b) Raman spectra, c–g) TG‐DSC curves, h) Nitrogen adsorption‐desorption isotherm, and i) Pore‐size distribution for CAs of different densities.

The X‐ray diffractometry (XRD) patterns shown in **Figure** **3**a reveal two broad peaks ≈26° and 43°, corresponding to the (002) and (100) planes of graphite, indicating a high degree of graphitization. The Raman spectra in Figure 3b exhibit characteristic D and G band peaks at 1330 and 1590 cm^−1^, respectively, indicating structural disorder and sp^2^‐bonded crystalline carbon. The intensity ratio of the D to G band (I_D_/I_G_), calculated using the Tuinstra‐Koenig equation, slightly increases from 1.14 to 1.17 with the increasing density of the CAs, indicating a consistent degree of graphitization.^[^ ^29^ ^]^ This consistency is further supported by the thermal weight loss profiles (Figure 3c–f). The TG‐DSC analysis, performed at a heating rate of 10 °C per minute under N_2_ protection, reveals that all CAs experience a rapid weight loss below 200 °C, attributed to the decomposition of impurities such as ZnCl_2_ and moisture content. The CAs demonstrate thermal stability up to the onset of an exothermic reaction occurring between ≈400 and 500 °C, suggesting that the porous network structure of CAs remains stable and operational below ≈400^ o^C. Carbon begins to decompose ≈400 ^ o^C in the air, undergoing oxidation reactions to produce carbon monoxide and carbon dioxide, thus exhibiting rapid weight loss reactions.

The N_2_ adsorption/desorption measurements provide insight into the pore‐size distributions of the CAs, as shown in Figure 3g,h. The adsorption/desorption isotherms exhibit characteristics of both type I and type IV isotherms, according to the IUPAC classification, indicating a continuous increase in adsorption capacity with increasing P/P_0_ value.^[^ ^26^ ^]^ Theoretically, the experimentally obtained isotherm represents the cumulative adsorption capacity, which should either remain constant or increase as pressure increases during the adsorption process. Conversely, during the desorption process, as pressure decreases, the adsorbed gas molecules gradually detach from the sample's surface. In the absence of surface tension effects, the adsorption and desorption curves would overlap. However, due to surface tension, molecules adsorbed at a certain pressure do not desorb at that same pressure. As pressure decreases further, adsorbed molecules continue to desorb, resulting in non‐overlapping adsorption and desorption lines and forming a hysteresis loop, as illustrated in Figure 3g. This hysteresis loop, observed between 0.4 and 0.95, is typically indicative of mesoporous materials. The pore sizes of CAs, primarily ranging from 3 to 5 nm as shown in Figure 3h, span the entire mesoporous range of 2–50 nm. It is also noteworthy that the CAs are relatively lightweight, attributed to the presence of extensive micrometer‐scale pores.

### 2.2 Tunable Epsilon‐Negative Response of Carbon Aerogels

**Figure 4:**
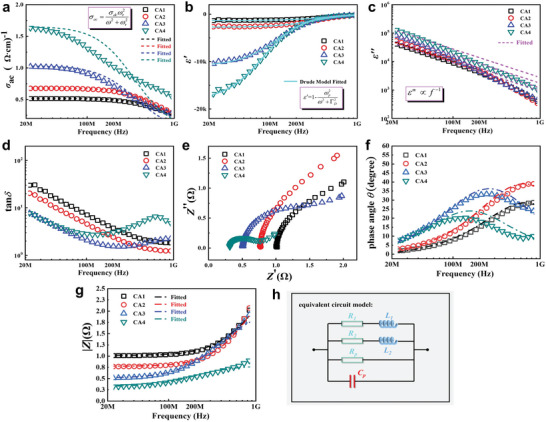
Frequency dependencies of the a) AC conductivity, b,c) Complex permittivity, d) Loss tangent, e) Reactance, f) Phase angle (*θ*), g) Impedance modulus (|*Z*|), and h) Circuit model for CAs.

The variable porosity of CAs naturally forms 3D conductive carbon networks, resulting in metal‐like conduction behavior (**Figure** **4**a). This behavior is characterized by a decreasing trend in alternating current (AC) conductivity (*σ_ac_*) with frequency, akin to the skin effect observed in metallic conductors. The skin depth (*δ*) can be expressed as:^[^ ^30^, ^31^ ^]^

(1) $$\delta =\sqrt{\frac{2}{\omega \mu \sigma_{dc}}}$$

where *σ_dc_* represents direct current (DC) conductivity, *ω* is the angular frequency, and *µ* denotes the static permeability, which remains constant for CAs. Thus, *δ* is inversely proportional to *ω* ^1/2^, leading to a decrease in *σ_ac_* at higher frequency regions. This trend becomes more pronounced with an increase in the density of CAs due to the enhanced 3D carbon network. The Drude model explains this phenomenon:^[^ ^32^, ^33^ ^]^

(2) $$\sigma_{ac}=\frac{\sigma_{dc}\omega_{\tau }^{2}}{\omega^{2}+\omega_{\tau }^{2}}$$

(3) $$\sigma_{dc}=\frac{Ne^{2}\tau }{m}=\frac{\omega_{p}^{2}\tau }{4\pi }$$

where *ω_τ_* (*ω_τ_* = 1/*τ*) is the relaxation rate and *ω*_p_ is the plasma frequency. The fitting results align well with the experimental data for CAs of lower density, while the fitting for CA4 deviates due to its heterogeneous composition and structure, which shows a more significant downward trend of *σ_ac_* than predicted by the Drude model. The skin effect refers to a phenomenon where the current distribution inside a conductor becomes uneven in alternating current or alternating electromagnetic fields. When there is alternating current or alternating electromagnetic field in a conductor, the current inside the conductor primarily concentrates on the surface layer of the conductor, that is, the current is concentrated on the thin layer on the surface of the conductor. The closer it is to the surface of the conductor, the higher the current density, while the actual current inside the conductor is smaller. This phenomenon leads to increased resistance of the conductor and higher power loss.

The engineered CAs exhibit an epsilon‐negative (*ε′* < 0) response across the 20MHz –1 GHz region, as depicted in Figure 4b (an enlarged version can be found in Figure S2, Supporting Information). The absolute value of *ε′* increases with the density of CAs from CA1 to CA4, due to the incremental low‐frequency plasmonic state. The dielectric constant (*ε* = *ε′−jε″*), indicative of a material's ability to store electric field energy, demonstrates that its negative value (*ε′* < 0) does not contravene the law of energy conservation. The epsilon‐negative response describes the motion state of free electrons, wherein the induced electric field within the material aligns with the external electric field, resulting in a negative dielectric constant. This harmonic motion of free electrons, termed plasma oscillation, is accurately described by the Drude model:^[^ ^34^, ^35^ ^]^

(4) $$\varepsilon^{\prime }=\varepsilon_{\infty }-\frac{\omega_{p}^{2}}{\omega^{2}+\Gamma_{D}^{2}}$$

(5) $$\omega_{p}=\sqrt{\frac{n_{eff}e^{2}}{m_{eff}\varepsilon_{0}}}$$

where *ε_∞_* is the optical limited permittivity (*ω* → ∞) that is typically assumed to be 1 at low frequencies, Γ*_D_* is the damping factor, *ω* is the external electric field, *n_eff_* is the effective electron density, and *m_eff_* is the electron mass. When *ω* is lower than *ω*_*p*_, the negative ε′ can be obtained. The CAs could also present metallic conductivity. As 3D carbon network formed in CAs, low‐frequency plasmonic oscillation occurred in composites, leading to well agreement of epsilon‐negative spectra with Drude model. The solid lines in Figure 4b represent fitting data by the Drude model, exhibiting high reliability.

The frequency dispersion of the epsilon‐negative response becomes more pronounced at higher CA densities due to an increasing trend of negative permittivity with frequency until reaching the so‐called cutoff frequency (*ω*_*p*_). Adjusting the porosity/density of CAs allows for tuning the concentration of free carriers, *ω*_*p*_, and oscillation strength, ultimately achieving a low‐dispersion epsilon‐negative response. The frequency dependences of *ε′* for the CA1 sample vary between ≈−1500 and −200, indicating a weakly and low‐dispersion epsilon‐negative response (0 < |*ε′*| <1000). For the CA2 sample, *ε′* values range between ≈−3000 and −500, with an incremental frequency dispersion. For the CA3 and CA4 samples, *ε′* values range between ≈−20000 and −400, marking a significant increase in magnitude.

The dielectric loss characteristics of CAs, represented by imaginary permittivity (*ε″*) and dielectric loss tangent (tan*δ* = |*ε″*/*ε′*|), are shown in Figure 4c,d. The CAs exhibit conduction loss (*ε*_c_ *″*) and relaxation loss (*ε*_r_ *″*) behavior across the entire tested frequency range due to the 3D carbon networks. Conduction loss dominates the dielectric loss, which can be described by the Debye theory:^[^ ^36^, ^37^ ^]^

(6) $$\varepsilon^{\prime \prime }={\varepsilon^{\prime \prime }}_{c}+{\varepsilon^{\prime \prime }}_{r}$$

(7) $${\varepsilon^{\prime \prime }}_{c}=\frac{\sigma_{dc}}{\omega \varepsilon_{0}}$$

(8)

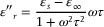

where *ε*_s_ and *τ* are static permittivity and relaxation time, respectively. The dashed lines represent fits to **Equation** (**7**), aligning well with measured data at low frequencies but deviating at higher frequencies. As we have explained in the AC conductivity spectrum, conductor materials will exhibit skin effect under high‐frequency electric field, leading to a deviation from the ideal model. Unlike the consistent decreasing trend over the 20MHz –1 GHz range for CA1 and CA2, the tan*δ* spectra for CA3 and CA4 samples exhibit noticeable variations at high frequencies (Figure 4d), attributed to the ENZ effect, accompanied by a change in loss mechanism and energy fluctuations.

### 2.3 Electrical Characteristics of Carbon Aerogels

CAs exhibiting an epsilon‐negative response demonstrate distinct electrical characteristics from conventional dielectrics with positive permittivity. The difference is detailed in Figure 4e–h. The relationship between the reactance (*Z″*) and the real part of the permittivity (*ε′*) for dielectrics can be described by the equation:^[^ ^38^, ^39^ ^]^

(9) $$\varepsilon^{\prime }=-\frac{Z^{\prime \prime }}{\omega C_{0}\left({Z^{\prime }}^{2}+{Z^{\prime \prime }}^{2}\right)}$$

where C_0_ is the vacuum capacitance, and *Z′* represents resistance. As shown in Figure 4e, all CA samples with negative permittivity exhibit positive values of *Z″*, indicating an inductive character. Typically, in conductor/insulator metacomposites, approaching but not exceeding the percolation threshold results in the conductive phase forming an inductor (*L*), while the isolated conductive phase acts as a capacitor (*C*), influencing the epsilon‐negative response through *LC* resonance. In porous CAs with 3D carbon networks, air serves as the insulating phase, and the CA itself as the conductive phase, allowing for simple tuning of the epsilon‐negative response strength by adjusting the CA's porosity/density.

Positive phase angle (*θ*) values indicate that the current phase lags behind the voltage phase across the 20 MHz–1 GHz region (Figure 4f). For CA1 and CA2 samples, which exhibit weakly and low‐dispersion epsilon‐negative response, the *θ* value increases across the entire tested frequency range, with peaks observed ≈200 MHz for CA3 and 100 MHz for CA4. This indicates more pronounced changes in the phase intensity of current and voltage with decreasing absolute values of *ε′* with frequency. Equivalent circuit analysis further clarifies the electrical characteristics of CAs, with the circuit model displayed in Figure 4h, and the dashed lines in Figure 4f representing fitting results. The impedance modulus (|*Z*|) spectra (Figure 4g), modeled by resistors (*R*_1_, *R*_2_, and *R_p_*), a capacitor (*C_p_*), and inductors (*L*_1_ and *L*_2_), match the experimental data of CAs well. The increase in |*Z*| values with frequency correlates with the skin effect at high frequencies, as discussed in the AC conductivity spectra, indicating the gradually weakening plasmonic state of CAs with frequency and resulting in increased impedance. The porous CAs we obtained has formed a 3D conductive network. This conductive network exhibits a low‐frequency plasma oscillation behavior under the excitation of an external alternating electric field, which is an inductive characteristic in terms of electrical characteristics. Therefore, we introduced a parallelly inductive components in the equivalent circuit. In addition, there is through air in the porous CAs, which shows capacitance. At the same time, we also introduced a resistance element to characterize the resistance characteristics at the interface between the electrode and the aerogel. The EM induction phenomenon becomes particularly noticeable with changes in the current passing through a conductor. For instance, utilizing a conductor to form a coil means that any alteration in the current flowing through this coil generates a pronounced EM induction effect. This self‐induced counter‐electromotive force within the coil acts to impede changes in current, thereby stabilizing it. More specifically, when an inductor is not carrying current, it will resist the onset of current flow upon circuit activation; conversely, if the inductor is conducting and the circuit is then opened, it seeks to keep the current flow constant. Leveraging the epsilon‐negative CAs developed in this study allows for the creation of inductors without the need for traditional winding. This aids in the miniaturization and integration of electronic components, presenting new opportunities for the design and development of more compact and efficient electronic devices.

### 2.4 Electromagnetic Simulation and Shielding Effectiveness of Carbon Aerogels

When electromagnetic radiation propagates through a plasma barrier, it interacts with the plasmonic state in CAs, which are excited by EM transverse waves. This interaction generates charge density fluctuations, leading to density EM oscillations akin to plasma.^[^ ^40^ ^]^ The characteristic frequency of these plasma oscillations is determined by the plasma structure and the effective concentration of free electrons within the CAs. The effectiveness of the plasma barrier in shielding against EM radiation is intricately linked to the frequency of the incident radiation. Radiation with frequencies below the plasma oscillation's characteristic frequency is gradually attenuated due to the excitation density of EM oscillation, resulting in either reflection or absorption. This attribute underlines the promising EM shielding capability of the plasma barrier.^[^ ^41^ ^]^

**Figure 5:**
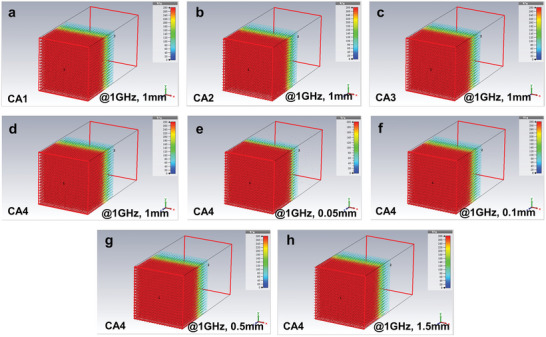
Electric field vector distributions in CAs at 1 GHz for different thicknesses. a) CA1, @1 GHz, 1 mm, b) CA2, @1 GHz, 1 mm, c) CA3, @1 GHz, 1 mm, d) CA4, @1 GHz, 1 mm, e) CA4, @1 GHz, 0.05 mm, f) CA4, @1 GHz, 0.1 mm, g) CA4, @1 GHz, 0.5 mm, h) CA4, @1 GHz, 1.5 mm.

**Figure 6:**
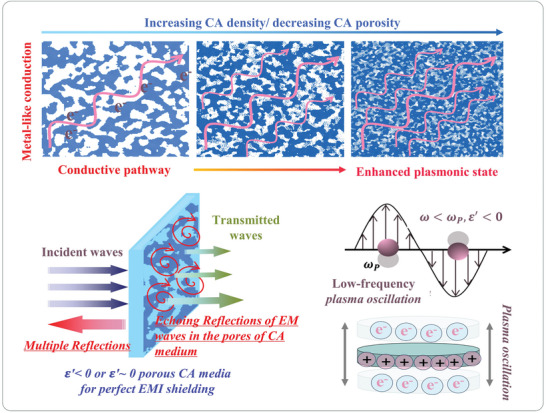
Schematic illustration of the generation mechanism for epsilon‐negative CAs and their promising EM shielding performance.

However, when the frequency of the incident EM radiation surpasses the characteristic frequency of stimulated EM oscillation, the plasma cannot be excited due to limitations in frequency response, rendering the plasma layer ineffective in shielding against high‐frequency.^[^ ^42^ ^]^ As demonstrated in **Figure** **5**a–h, layers of CAs exhibiting an epsilon‐negative response maintain excellent shielding effectiveness at 1 GHz, regardless of variations in thickness from 0.05 to 1.5 mm. For comparison, electric field vector distributions in CAs at 20 MHz for thickness of 1 mm are presented in Figure S3 (Supporting Information). Moreover, the reflectivity spectra of CAs with different densities and thicknesses given by Computer Simulation Technology (CST) software are shown in Figure S4 (Supporting Information). This consistent effectiveness can be attributed to two primary mechanisms: the reflection of most incident EM waves by the CAs, facilitated by their plasmonic oscillation state, and the multiple reflection losses within the CA's porous structure for any penetrating EM waves. These mechanisms collectively ensure superior EM shielding effectiveness, as depicted schematically in **Figure** **6**. The utilization of lightweight CAs, as developed by this study, for EM shielding materials offer significant advantages.^[^ ^40^, ^41^, ^42^, ^43^ ^]^ These advantages include effective protection for electronic devices and their surroundings, prevention of EM information leakage, interruption of EM wave propagation paths, and suppression of EM radiation and interference. This approach is anticipated to lay a foundational basis for addressing challenges associated with EM radiation and interference, thereby contributing to the advancement of research in EM shielding solutions.^[^ ^44^, ^45^, ^46^, ^47^ ^]^

## 3 Conclusion

This study presents an efficient, low‐cost method for fabricating CAs without the need for rigorous drying conditions. By precisely adjusting the density/porosity of CAs, a tunable epsilon‐negative response is achieved in the RF region, stemming from the intrinsic plasmonic state of the 3D carbon network. The *ε′* of these CAs ranges from ≈−1500 to −200, showcasing a weakly and low‐dispersion epsilon‐negative response. Throughout the entire tested frequency spectrum, the epsilon‐negative CAs exhibit inductive characteristics, indicating their unique electric behavior. EM simulations conducted as part of this study highlight the substantial EM shielding capabilities of the CAs, demonstrating their potential as effective EM shielding materials. This work is expected to advance the development and application of EM metamaterials, as well as the recycling of lignin waste. The process outlined is economically viable, efficient, and environmentally friendly, involving the conversion of lignin into lightweight CAs that exhibit an epsilon‐negative response.

## 4 Experimental Section

### Synthesis of Carbon Aerogels with Varied Porosity

Initially, ZnCl_2_ and lignin experienced thoroughly mixing process with the CH_2_O solution until a brown viscous slurry formed. It was worth noting that the soda lignin powders were sourced from Shandong Longlive Co., Ltd. Analytical grade ZnCl_2_ and CH_2_O solution (37–40%) were acquired from Beijing Chemical Works and utilized without modification. Here, ZnCl_2_ simultaneously acted as ideal pore forming agents, activators, and hard templates to achieve different porosity of CAs. The mixtures were then putted into the Teflon‐lined autoclave and heated at 160 °C for 2 h before being vacuum dried. The carbonization process at 900 °C for 2 h under an N_2_ atmosphere produced CAs with varying porosity and density. These CAs were further polished and shaped into discs, each 2 mm thick, for dielectric testing and characterization. The resulting CAs were designated CA1 (density: 0.456 g cm^−3^), CA2 (density: 0.517 g cm^−3^), CA3 (density: 0.609 g cm^−3^), and CA4 (density: 0.775 g cm^−3^). Noteworthily, aerogels typically refer to porous materials with relatively low density, ≈10 mg cm^−3^.^[^ ^28^, ^36^ ^]^ However, the aerogel developed in this study has a density of 500 mg cm^−3^. This discrepancy arises from this focus on lightweight structures compared to previously reported metamaterials utilizing functional phases such as metals, GR, and CNTs.^[^ ^29^, ^30^, ^31^ ^]^ Therefore, the term “aerogels” is continued to be used until a more appropriate alternative is identified.

### Measurement and Characterization

The structural morphology of the CAs was examined using scanning electron microscopy (SEM, SU‐70) and high‐resolution transmission electron microscopy (HRTEM, JEOL‐1230). The degree of graphitization was determined by X‐ray diffractometry (XRD, X'Pert Pro) and Raman spectroscopy (Jobin‐Yvon HR800). Thermal stability assessments were conducted using thermal gravimetric and differential scanning calorimetry (TG‐DSC, STA499F3, Netzsch). Porosity and pore size were evaluated using N_2_ sorption measurements on an Autosorb‐1 (Quantachome, USA), with data analyzed via Barrett‐Emmett‐Teller (BET) calculations and the Barrett‐Joyner‐Halenda (BJH) model. Electrical properties such as complex permittivity (*ε′*, *ε″*), AC conductivity (*σ_ac_*), and impedance (|*Z*|, *Z″*) were measured with an Impedance Analyzer (Agilent E4991A) using a 16453A fixture following standard procedures.^[^ ^36^ ^]^ Finally, simulations of the electric field vector distribution in CAs at 1 GHz with varying thicknesses were conducted using CST software.

## Conflict of Interest

The authors declare no conflict of interest.

## Supporting information

Supporting Information

## Data Availability

The data that support the findings of this study are available from the corresponding author upon reasonable request.
