# Supplementary material for: Lignin‐Derived Lightweight Carbon Aerogels for Tunable Epsilon‐Negative Response
Source: Adv Sci (Weinh). 2024 May 7;11(26):2401767. doi: 10.1002/advs.202401767 (PMC11234391; doi:10.1002/advs.202401767)
Supplement: Supplementary file 1 — Supporting Information [file ADVS-11-2401767-s001.docx]

Supporting Information

Lignin-Derived Lightweight Carbon Aerogels for Tunable Epsilon-Negative Response

*Yunpeng Qu, Yunlei Zhou, Qiuyun Yang, Jun Cao, Yao Liu*, Xiaosi Qi*, and Shan Jiang**

*Corresponding authors

[liuyao@sdu.edu.cn](mailto:liuyao@sdu.edu.cn) (Y. Liu);

[xsqi@gzu.edu.cn](mailto:xsqi@gzu.edu.cn) (X. Qi);

[jiangshan@xidian.edu.cn](mailto:jiangshan@xidian.edu.cn) (S. Jiang)

**
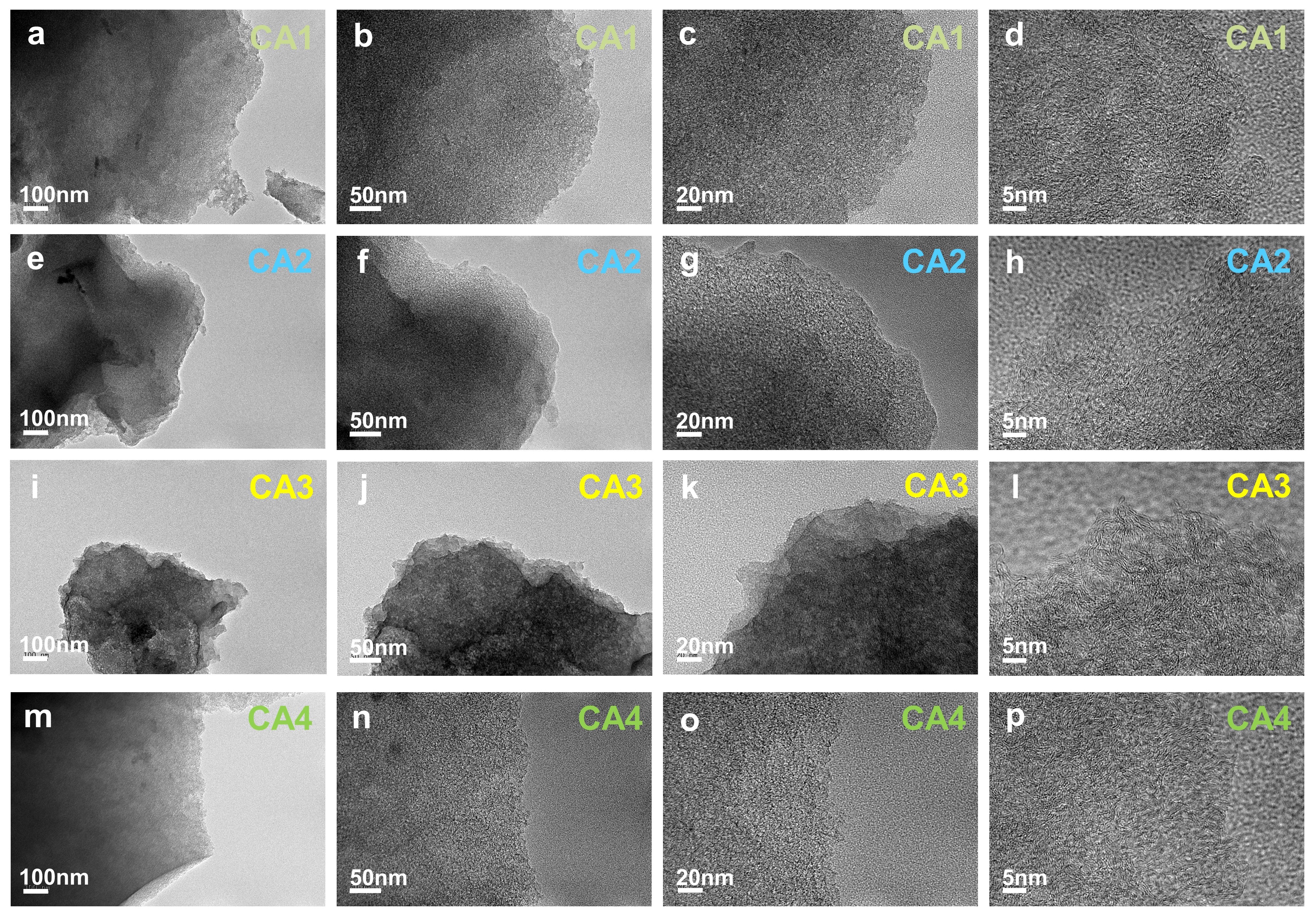
**

**Figure S1** TEM and HRTEM maps of CAs with varying densities.

**
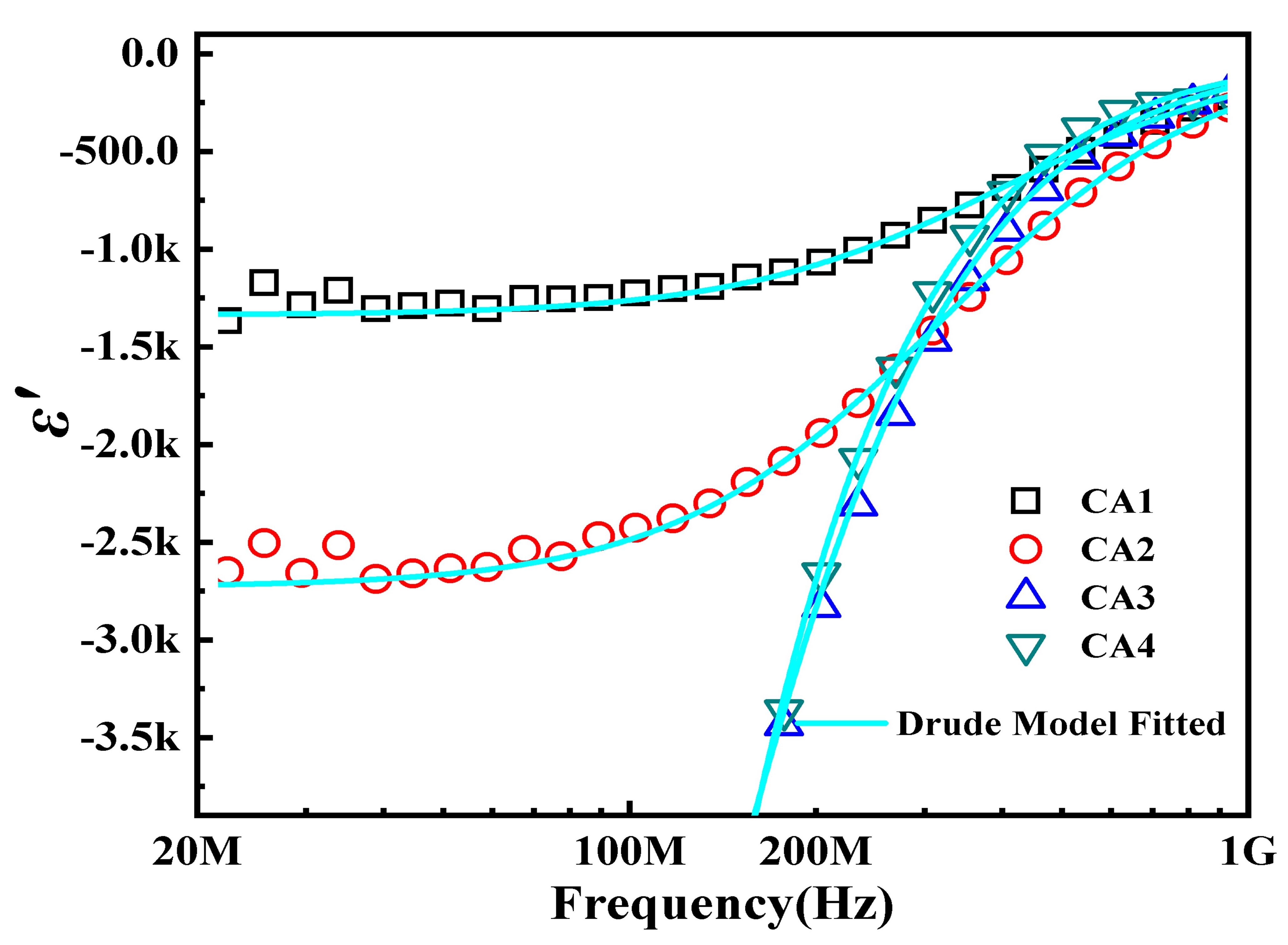
**

**Figure S2** Frequency dependencies of the real permittivity for CAs.

**
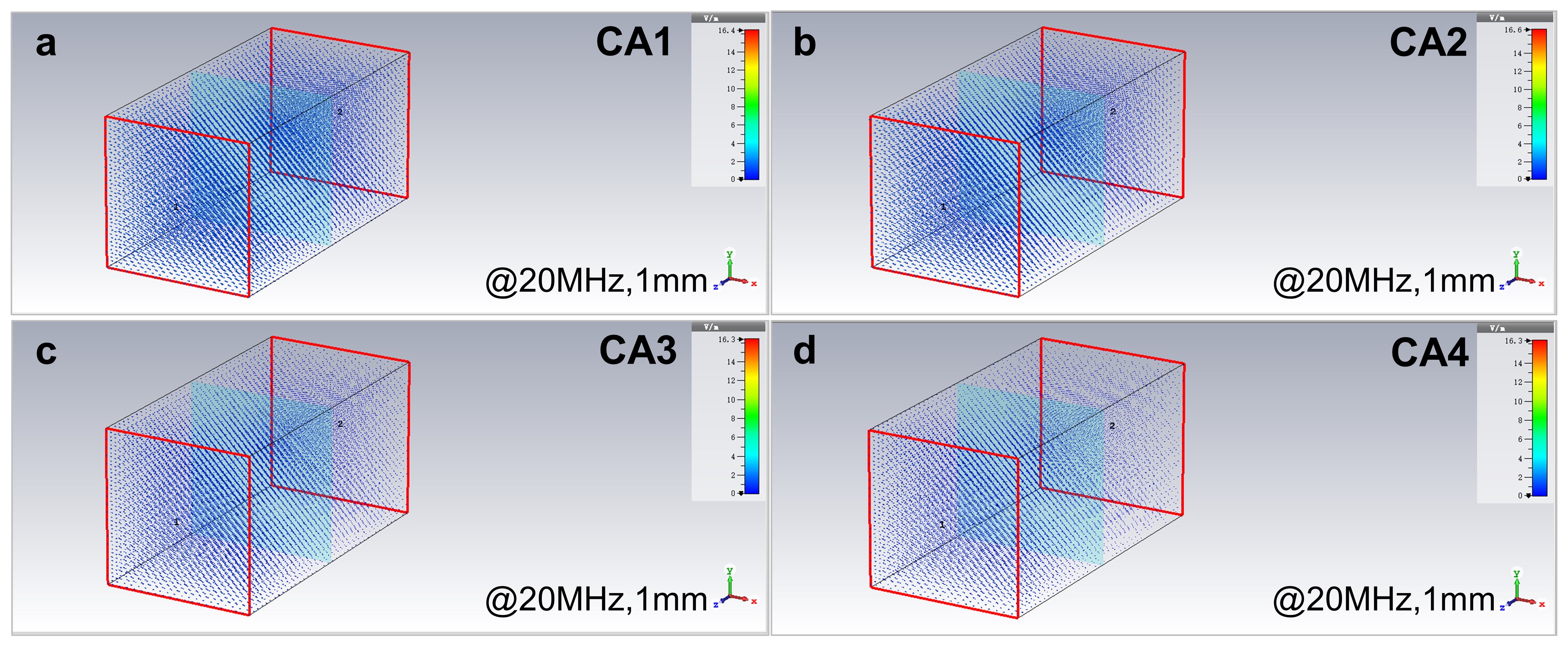
**

**Figure S3** Electric field vector distributions in CAs at 20 MHz for thickness of 1 mm.

**
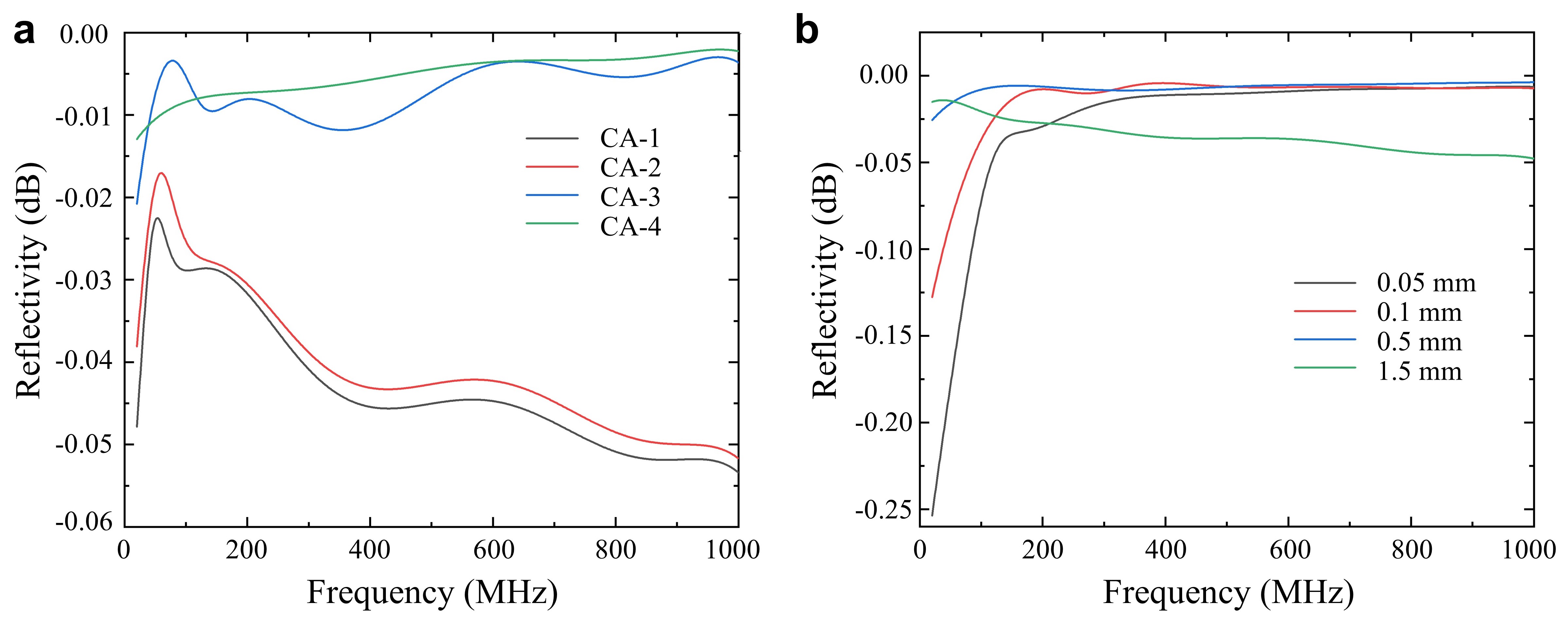
**

**Figure S4** Reflectivity spectra of CAs with **a)** different densities and **b)** thicknesses given by CST software.
